# Factors associated with child malnutrition in mountainous ethnic minority communities in Lao PDR

**DOI:** 10.1080/16549716.2020.1785736

**Published:** 2020-08-03

**Authors:** Sayvisene Boulom, Dirk R. Essink, Myung-Hee Kang, Sengchanh Kounnavong, Jacqueline E.W. Broerse

**Affiliations:** a Rural Economic and Food Technology Department, Faculty of Agriculture, National University of Laos, Vientiane, Lao PDR; b Athena Institute, Faculty of Science, Vrije Universiteit, Amsterdam, Netherlands; c Lao Tropical and Public Health Institute, Vientiane, Lao PDR

**Keywords:** LEARN: Sexual Reproductive Health, ANC and Nutrition, Malnutrition, wasting, stunting, dietary diversity

## Abstract

**Background:**

Although in many low- and middle-income countries undernutrition is steadily decreasing, nutritional challenges persist in remote communities, such as those in mountainous areas of Lao PDR. Isolated, with limited access to food and to health care and other public services, local diets are low in both quantity and diversity. Data needed to guide policy and planning are lacking.

**Objectives:**

The study aimed to identify the extent of malnutrition and associated factors among children aged 12–47 months in remote mountainous communities in Lao PDR.

**Methods:**

A cross-sectional survey was conducted in Nong district, Savannakhet province, covering 173 households, involving heads of households, mothers and their children aged 12–47 months.

**Results:**

The prevalence of undernutrition was very high among the 173 children studied: 72.8% were stunted, 50.3% underweight and 10.4% wasted. Key factors showing significant positive associations with nutritional status were assets (mobile phone or electric rice mill), collection of non-timber forest products, and household dietary diversity. Negative associations were found with experience of malaria and consuming high amounts of white roots and tubers. Immediate causes of malnutrition were prevalent; half the children had insufficient consumption of all micro- and macronutrients. Diseases were highly prevalent; 30% had recently experienced fever. The households in these villages were quite homogeneous. All scored high on causes of malnutrition; 90% of households experienced food insecurity, nearly all lived below the poverty line, and almost two-thirds of household heads and nearly all mothers had had no education.

**Conclusion:**

This study identified multiple factors influencing child malnutrition, including low household food diversity, food insecurity, and poor feeding practices among ethnic minority people living in a difficult environment with limited resources. Child undernutrition in these poor communities is complex. Interventions are needed in different sectors, including agricultural production, knowledge on feeding and health services.

## Background

Malnutrition is a serious global health problem [1]. Low- and middle-income countries (LMICs) have been mostly faced with undernutrition, including underweight, wasting and stunting, while overweight and obesity have been greater challenges in developed countries. Yet, many LMICs are experiencing what is known as the double burden of malnutrition, where both undernutrition and overweight are prevalent [2]. Child malnutrition has been a serious cause of mortality and morbidity worldwide [3]. Malnutrition during the early development of children – especially during the first thousand days – has negative effects on health, economic and educational performance in their future life [4,5].

International organizations and local governments have put reduction of child malnutrition high on their policy agendas, and globally, improvements have been made [6]. The World Health Organization [7] estimated that global prevalence of stunting in children under five decreased from 32.5% in 2000 to 21.9% in 2018; the prevalence of wasting was reduced from 11% in 1999 [8] to 7.3% in 2018 [7]. Also in Lao PDR, the setting of this study, concerted government actions and rapid economic growth have contributed to reductions in undernutrition according to anthropometric indicators. For example, between 2015 and 2017 stunting was reduced from 44% to 33%. underweight from 35% to 27%, and wasting from 9.6% to 9% [9]. However, national statistics tend to overlook areas where poor nutritional status persists. Field reports suggest that in Lao PDR as in other countries, the remote mountainous areas with ethnic minority populations are likely to be one of these under-reported areas [10,12].

A range of causes contributes to malnutrition among children [13]. *Immediate causes* of malnutrition are inadequate food intake and diseases. Dietary diversity is positively associated with nutrient intake and nutritional status of children [14]. *Underlying causes* have been identified as household food insecurity, inappropriate care and feeding practices, unhealthy household environment, and inadequate health services [13]. For example, household food insecurity was significantly associated with children’s diarrhoea, respiratory diseases and parasite infections in Colombia [15]. More *basic causes* are related to human, economic and organizational resources [13]. For example, mothers with lower education and lower household income have been shown to provide lower dietary diversity for their children in Ethiopia and China [16,17]. These factors plus household food insecurity were associated with malnutrition in Bangladesh, Ethiopia and Vietnam [18] while a study in Colombia demonstrated that the combination of malaria, food insecurity and low socio-economic status led to child malnutrition [19].

Rural and mountainous communities in LMICs are particularly prone to many of the underlying and basic causes of malnutrition described above. These communities are often isolated with limited access to food, health care, education, markets, and other public services. Access to clean water for consumption and daily use is a challenge. Subsistence farming plays an important role in these communities’ livelihood [20]. Non-timber forest products (NTFPs) are alternative food sources and can potentially contribute to food security, nutrition and livelihood in poor rural areas [21,24]. However, there is still little knowledge about the link between NTFPs and nutritional status of children. Given the complexity of the problem of malnutrition, particularly in rural, remote areas in LMICs, policies and interventions require a thorough understanding of not only the immediate causes, but also the underlying and basic causes.

Based on field reports and the scarce scientific evidence, we assume that in Lao PDR there are pockets where malnutrition is much higher than the averages provided by national statistics. These areas hardly benefit from national policies targeting nutrition, and risk falling even further behind in socio-economic development. Demonstrating the inequitable distribution of poor nutritional status is the first step in solving it. Therefore, this study aimed to provide insights into the prevalence and associated factors of malnutrition among remote upland ethnic minorities in Nong district, Savannakhet Province, Lao PDR, in order to contribute to evidence-informed, contextualized policies and interventions.

## Methods

### Setting, study design and data sources

A cross-sectional survey was conducted in Nong, one of the poorest mountainous districts, in Savannakhet province. Twenty-three villages were selected for the survey, all located in isolated areas and difficult to access due to mountainous and rocky roads; many villages were cut off from the outside world during the rainy season.

### Study population

The study applied randomized and stratified sampling methods. The study population was the total population in the 23 villages. Using a standard sample size formula set at 95% confidence level and confidence interval of 5, we wanted to recruit 296 households. We used random selection to identify 15 households per village, resulting in 345 households, from which we selected the 173 households that had a father, a mother and at least one child between 12 and 47 months of age. During a first round of selection, we had planned to include households with children under 5 years, but the numbers of children below 12 months and above 47 months were too low to apply statistical analysis and were excluded from further analysis.

### Measurement of variables

Data were collected using questionnaires, anthropometric measurements, and 24-hour food intake recall as described below.

*Questionnaires*: The head of the household was asked about the socio-economic status of the household and their livelihood. Mothers were asked about the dietary intakes of family members, including children, and about care practices. Household dietary diversity score (HDDS) was classified using 12 food groups. The minimal dietary diversity scores for children (MDDS) were determined using seven groups [25]; adequate MDDS for children is considered to be four [26]. Food insecurity experience scale (FIES) was used to determine the severity of food insecurity of households or individuals based on 8 questions regarding their access to adequate foods. The severity was classified into three levels, namely mild (<4 raw scores), moderate (4, 5 or 6 raw scores) and severe food insecurity (7 or 8 raw scores).

*Anthropometric measurements* were applied to evaluate the nutritional status of the children aged between 12 and 47 months. After finishing the interviews, the mothers took their children to measurement points where trained public health staff measured the weight and height. The staff used weighing scales for SECA 874 U and measured children’s recumbent length (SECA 417) and height (SECA 213).

*24-hour food intake recall*: Investigation of daily dietary intakes of children used the FAO 24-hour recall questionnaire, asking mothers about estimated quantities using a food picture book showing food portion size, popular local menus, and quantities of ingredients [27].

Nutritional status of children was defined according to WHO standards [28] as:

- Stunting: moderate and severe: height-for-age Z-score between −2SD to −3 SD and <−3 SD, respectively, from the median of WHO reference population;
- Wasting: Moderate and severe: weight-for-height between −2 SD to −3 SD and <−3 SD, respectively, from the median of WHO reference population;
- Underweight: weight-for-age Z-score between −2SD to −3 SD and <−3 SD, respectively, from the median of WHO reference population.

### Statistical analysis

IBM SPSS version 23 was applied for statistical evaluation. All surveyed data were entered into Epidata software. Nutrient intake was managed with the software ‘INMUCAL version 3.2’. Each nutrient value was compared with the Thai dietary recommended intakes; then nutrient adequacy rate was used to assess individual dietary intake. The Thai Dietary Reference Intake (DRI) was used because Laos currently does not have recommended amounts, while its lifestyle and culture are similar to those in Thailand. The Thai DRI has four concepts: Estimated Average Requirement (EAR), Recommended Dietary Allowance (RDA), Adequate Intake (AI) and Tolerable Upper Intake Level (UL) [29]. The EAR concept is used to determine whether or not nutrients are consumed but is not set; a problem of judgment arises when nutrition is judged using % DRI. Therefore, levels were determined by dividing RI into 50% DRI, 75% DRI, and 100% DRI.

The chi-square test was performed to determine the association between categorical variables. When dependent variables were continuous, means from two groups were tested by Bivariate test. In addition, odds ratios were used to estimate the risk ratio between groups and to test factors potentially associated with nutritional status.^1^1 US dollar = 8800 kip.

## Results

The analysis included data from 173 households of which the socio-economic characteristics are shown in Table 1. Almost two thirds of household heads and nearly all mothers had no education. Almost all households practiced subsistence farming and also provided workers for other farms. The two main ethnic groups in the study area were Mang-kong (73%), and Ta-oy (27%). Most households (85%) consisted of 5–10 family members; 82% of households were very poor, having an annual income of less than 3,000,000 Lao kip per year (under 1 US dollar per day).

**Table 1.** Socio-economic characteristics of 173 households in remote mountainous areas of Lao PDR.

Variables at household level (*n* = 173)	Numbers	Percentage
Household’s head age		
15–25	15	9
26–45	106	61
>45	52	30
Mother’s age		
15–25	38	22
26–45	120	69
>45	15	9
		
Education level of household’s head		
No schooling	111	64
Primary level	56	32
Secondary level	4	2
Higher level	2	1
Occupation of household head		
No occupation	25	15
Family worker (non-wage)	68	39
Worker in farms	72	41
Construction worker	2	1
Education level of mother		
No schooling	157	91
Primary level	14	8
Secondary level	2	1
Higher level	0	0
Occupation of mother		
No occupation	24	14
Family worker (non-wage)	54	31
Farm worker	54	31
Housewife	31	18
Construction worker	2	1.2
Others	1	0.6
Ethnicity		
Mang-kong	127	73.4
Ta-oy	46	26.6
Number of people in households		
1–4	16	9.2
5–10	147	85
>10	10	5.8
**Household annual income**	***N*** = 152	
≤3.000.000 kip^1^	124	82
>3.000.000 kip	28	16
**Agricultural production diversity**	Mean = 4.2	
<3 types	31	17.9
3 – <4 types	30	17.3
4 – <5 types	42	24.3
≥5 Types	70	40.5
**Household wealth index**	***N*** = 171	
Poor	68	39.3
Middle	**35**	20.2
Richest	**68**	39.8
**Harvesting non-timber forest products**	***N*** = 171	
<3 NTFP	71	42
3–7 NTFP	96	56
>7	4	2
**Food insecurity experience scale**	***N*** = 173	
Food security	17	9.8
Mildly food insecurity	53	30.6
Moderated food insecurity	68	39.3
Severe food insecurity	35	20.2
**Distance to Market**		
≤40 km	21	12.1
>40 km	152	87.9
**Variables for children (12–47 months)**	***N*** = 173	
Male	84	48.6
Female	89	51.4
Core indicators for IYCF		
Breastfeeding within 1 hour of birth	160	92.5
Exclusive breastfeeding children under 6 months	11	6
Introduction of solid and semi-solid foods		
<7 Days after birth	55	32
≥1 week and <1 month after birth	28	16
≥1 Month and <6 months after birth	56	32
Don’t know	34	20
Types of first supplementary feeds		
Cow milk	1	0.6
Infant formula	2	1.2
Rice water	136	78.6
Pre-chewed rice	27	15.6
Don’t know	3	1.7
Minimum dietary diversity for children		
Less than 4 food groups	92	53
More than 4 food groups	81	47
Disease in last 2 weeks		
Diarrhea	35	20.2
Cough/Respiratory problems	19	11
Fever	56	32.4
Disease last 12 months		
Malaria	61	35

IYCF: Infant young child feeding practices.

### *Nutritional status of children between 12*–*47 months*

**Figure 1.**
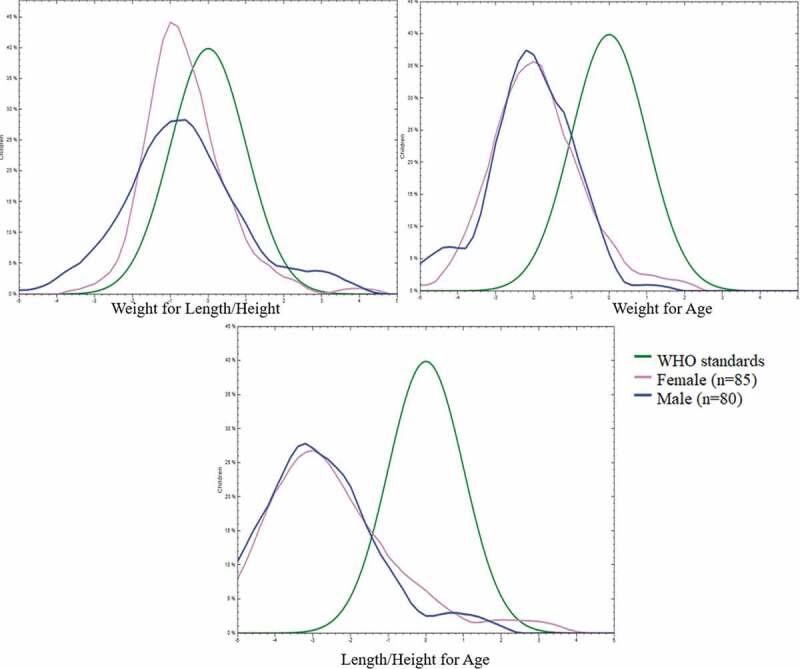
Comparison of nutritional status in mountainous villages of Lao PDR and WHO standards.

The prevalence of wasting was 10.4%, underweight was 50.3%, and stunting 72.8% (Table 2). Figure 1 shows that all indicators were clearly below the WHO standards. Weight-for-height (wasting) was closest to WHO standards, whereas especially stunting, and, to a lesser degree, wasting were far below WHO standards. Weight-for-age and height-for-age of boys and girls were similar, both far below the WHO standards.

### Underlying factors of child malnutrition

We investigated potential factors underlying the high prevalence of malnutrition. Food insecurity remains highly persistent in both Mang-kong and Ta-oy communities: 90% of households had experienced episodes of food insecurity, with 20% of households facing severe food insecurity (Table 1). Our results show that access to food is likely to be problematic as access to markets was poor and production diversity is low. Households on average produced fewer than four food groups (crops and animal products), and almost half of respondents produced three or less food groups. Market access was poor because 88% of households are located more than 40 km from a market. Non-timber forest products provide an alternative food source; 56% of people collected between four and seven types of foods from the forest.

With regard to food consumption by young children, patterns were revealed that could help to explain malnutrition. For example, although nearly all mothers (92%) started breastfeeding within one hour after giving birth, only 6% of children were exclusively breastfed during the first 6 months. Almost one third of mother introduced supplementary semi-solid and solid foods after a few days or months. Rice water (79%) and pre-chewed rice (16%) were common types of first semi-solid and solid additions.

Just under half of the children studied (47%) were able to meet the recommended minimum MDDS of four food groups per day. Diseases were also a barrier to good nutrition; 32% of the children had experienced fever, 20% had had diarrhea, and 11% had been affected by cough and respiratory issues during the last 2 weeks. In addition, 35% of children had been infected by malaria in the past year.

### Dietary nutrient intake adequacy

The children in our study were found to have multiple micronutrient deficiencies (Table 3). Their nutrient intakes were compared with Thai dietary reference intakes (DRI). Several micronutrient intakes of the children 12–47 months were lower than 50% of the recommended nutritional values. The serious deficiencies of micronutrients included calcium (156 children out of 173), vitamin A (108 children), thiamine (111 children) and vitamin C (80 children) (Table 3). These children had a high probability of malnutrition due to multiple micro-nutrient deficiencies. However, certain children did consume sufficient protein, niacin, iron, riboflavin and energy. The proportions of children who achieved 100% of recommended doses were: 47% for protein, 46% for niacin, 27% for iron, 24% for riboflavin and 22% for energy. Although we tested for association of these intake inadequacies with malnutrition we did not find any significant relationships among them.

**Table 2.** Nutritional status of children 12–47 months (*N* = 173).

	Wasting (WHZ)		Stunting (HAZ)			Underweight (WAZ)
	Male (%)	Female (%)	Male (%)	Female (%)	Male (%)	Female (%)
Normal	70 (83.3)	85 (95.5)	20 (23.8)	27 (30.3)	42 (50.0)	44 (49.4)
Moderate	9 (10.7)	3 (3.4)	24 (28.6)	22 (24.7)	29 (34.5)	32 (36.0)
Severe	5 (6.0)	1 (1.1)	40 (47.6)	40 (44.9)	13 (15.5)	13 (14.6)
Total (%)	18 (10.4)		126 (72.8)		87 (50.3)

**Table 3.** Number (%) of children aged 12–47 months who consumed the nutrients below the 50%, 50~74.9%, 75~99.9% and over the 100% of the Thai DRI (*N* = 173).

	Mean ±SD	DRI	Under 50%	50%~74.9%	75%~99.9%	Over 100%
Energy	723 ± 28.4	1000	52 (30.0%)	53 (30.6%)	30 (17.3%)	38 (21.9%)
Protein	23 ± 1.3	18	22 (12.7%)	40 (23.1%)	29 (16.8%)	82 (47.4%)
Calcium	136 ± 14.4	500	156 (90.1%)	8 (4.6%)	6 (3.5%)	3 (1.7%)
Iron	4.13 ± 0.2	5.8	82 (47.4%)	31 (17.9%)	14 (8.1%)	47 (27.2%)
Vitamin A	144 ± 24	400	108 (62.4%)	17 (9.8%)	20 (11.6%)	9 (5.2%)
Thiamin	0.3 ± 0.05	0.5	111 (64.2%)	26 (15.0%)	24 (13.9%)	12 (6.9%)
Riboflavin	0.4 ± 0.02	0.5	70 (40.5%)	33 (19.1%)	29 (16.8%)	41 (23.7%)
Vitamin C	16 ± 1.9	40	80 (46.2%)	19 (11.0%)	18 (10.4%)	16 (9.2%)
Niacin	6.7 ± 0.3	6	37 (21.4%)	44 (25.4%)	12 (6.9%)	80 (46.2%)

SD: standard deviation, DRI: dietary recommendation intake.

### Factors associated with child nutritional status

A number of factors were found to be associated with child nutritional status (Tables 4–6). However, a number of expected associations could not be established. For example, total wealth index, educational attainment and the specific nutrient intakes were not significantly associated with any of the nutritional outcomes. Certain household assets were, however, associated with wasting. Perhaps surprisingly, there was a higher proportion of children with wasting in households with a radio compared to those without one (Table 4). The difference however was only just significant, and may have been influenced by the relatively low number of households having radios (21/173). Stunting was associated with a lack of adequate nutrients. Absence of a rice mill was associated with higher levels of stunting (Table 5). Household assets, collecting non-timber forest products (NTFPs), and household dietary diversity were significantly correlated with children being underweight. Children from households without a mobile phone and without rice mills had a higher prevalence of underweight than those with these assets. Children from households that gathered insects and wild eggs such as ant eggs also had lower prevalence of underweight, compared to these who did not (Table 6). Our data revealed that children from households that had HDDS with high proportions of white roots, tubers and plantain had a greater of being underweight, compared to other households that did not include so much of the white roots in their diet. Many households had experienced food insecurity because of rice shortage, so other carbohydrate sources had to fill the gap.

**Table 4.** Prevalence of wasting and its odds ratio.

Independent variables			Wasting (*N* = 173)				
			Yes	No	Total	OR (95% CI)	*p*-Value
Wealth Index	Poor		9	59	68	1	–
	Middle		2	33	35	0.397 (0.08–1.95)	0.255
	Rich		6	62	68	0.634 (0.21–1.89)	0.414
	Unknown				2		
Household assets	Radio	Yes	5	16	21	0.299 (0.09–0.95)	0.048*
		No	13	139	152		
	Mobile phone	Yes	6	83	89	2.3 (0.82–6.45)	0.13
		No	12	72	84		
	Rice mill	Yes	1	18	19	2.10 (0.26–16.8)	0.7
		No	16	137	153		
		Unknown			1		
Non-timber forest products	<3 types		9	62	71	1	
	4–7 types		9	87	96	0.71 (0.27–1.89)	0.48
	>7 types		0	4	4	0 (0)	0.99
	Unknown				2		
	Gather insects	Yes	4	62	66	0.42 (0.32–1.33)	0.2
		No	14	91	105		
		Unknown			2		
	Gather ant eggs	Yes	1	10	11	0.84 (0.1–6.98)	0.87
		No	17	143	160		
		Unknown			2		
HDDS	<3 Low		1	24	25	1	
	4–6 Medium		9	70	79	3.08 (0.37–25.6)	0.29
	7–9 Good		7	53	60	3.17 (0.36–27.2)	0.29
	10–12 well		1	8	9	3.00 (0.17–53.7)	0.45
HDDS1	Cereal group	Yes	16	116	155	2.69 (0.59–12.2)	0.24
		No	2	39	18		
HDDS2	White roots and tubers group	Yes	14	94	108	2.27 (0.71–7.22)	0.2
		No	4	61	65		
HDD12	Spices, condiments and pre-packed foods	Yes	14	126	140	0.80 (0.25–2.62)	0.75
		No	4	29	33		
FIES	Food security		2	15	17	1	-
	Mild		8	45	53	1.33 (0.26–6.98)	0.73
	Moderate		6	62	68	0.72 (0.13–3.96)	0.71
	Severe		2	33	53	0.46 (0.06–3.54)	0.45
MDDS-Children	<4 food groups		8	73	81	1	
	≥ 4 food groups		10	82	92	0.89 (0.33–2.39)	0.831
Breastfeeding the first one hour		Yes	16	144	13	0.61 (0.12–3.00)	0.62
		No	2	11	160		
Exclusive breastfeeding for 6 months	Yes	Yes	0	11	11	0.92 (0.88–0.97)	0.6
		No	18	143	161		
		Unknown			1		
First time introduction supplementary foods after birth	≥1 Month and <6 months		4	52	56	1	
	<7 Days		4	51	55	1.02 (0.24–4.29)	0.97
	≥1 week and <1 month		5	23	28	2.83 (0.69–11.5)	0.15
	Don’t know		5	29	34		
Child diseases last two weeks	Diarrhea	Yes	6	29	35	2.58 (0.83–8.05)	0.10
		No	8	100	108		
		Unknown			30		
	Fever	Yes	4	52	56	0.56 (0.16–1.91)	0.40
		No	10	74	84		
		Unknown			33		
	Cough and respiratory issue	Yes	2	17	19	1.03 (0.21–5.07)	0.96
		No	12	106	118		
		unknown			36		
Malaria last year	Normal		5	79	84	1	
	Malaria		9	52	61	2.73 (0.89–8.62)	0.086
	Don’t know		4	24	28		

*P < 0.05. HDDS: household dietary diversity score. FIES: food insecurity experience scale. MDDS-Children: minimum dietary diversity score for children.

**Table 5.** Prevalence of stunting and its odds ratio.

			Stunting				
Independent variables			Yes	No	Total	OR (95% CI)	*p*-Value
Wealth Index	Poor		20	48	68	1	–
	Middle		6	29	35	2.014 (0.73–5.59)	0.18
	Rich		21	47	68	0.933 (0.45–1.94)	0.85
	Unknown				2		
Household assets	Radio	Yes	19	2	21	0.25 (0.05–1.12)	0.06
		No	107	45	152		
	Mobile phone	Yes	63	26	89	1.23 (0.63–2.42)	0.6
		No	63	21	84		
	Rice mill	Yes	10	9	19	2.72 (1.03–7.2)	0.038*
		No	115	38	153		
		Unknown			1		
N-timber forest products	<3 types		53	18	71	1	
	4–7 types		69	27	96	0.87 (0.43–1.74)	0.69
	>7 types		2	2	4	0.34 (0.05–2.59)	0.29
	Unknown				2		
	Gather insects	Yes	46	20	66	0.79 (0.4–1.58)	0.59
		No	78	27	105		
		Unknown			2		
	Gather ant eggs	Yes	5	6	11	0.43 (0.13–1.47)	0.17
		No	118	42	160		
		Unknown			2		
HDDS	<3 Low		19	6	25	1	
	4–6 Medium		59	20	79	0.93 (0.32–2.65)	
	7–9 Good		43	17	60	0.79 (0.27–2.34)	
	10–12 well		5	4	9	0.39 (0.08–1.96)	
HDDS1	Cereal group	Yes	93	39	132	0.58 (0.24–1.36)	0.23
		No	33	8	41		
HDDS2	White roots and tubers group	Yes	14	94	108	1.91 (0.97–3.78)	0.077
		No	4	61	65		
HDD12	Spices, condiments and pre-packed foods	Yes	98	42	140	0.48 (0.15–1.15)	0.12
		No	28	5	33		
FIES	Food security		14	3	17	1	-
	Mild		42	11	53	0.81 (0.19–3.36)	0.78
	Moderate		47	21	68	0.48 (0.12–1.84)	0.28
	Severe		23	12	35	0.41 (0.09–1.71)	0.22
MDDS-Children	<4 food groups		56	25	81	1	
	≥4 food groups		70	22	92	0.7 (0.36–1.38)	0.3
Breastfeeding the first one hour		Yes	117	43	160	1.2 (0.35–4.13)	0.75
		No	9	4	13		
Exclusive breastfeeding for 6 months		Yes	9	2	11	1.69 (0.35–8.14)	0.72
		No	117	44	161		
		Unknown			1		
First time introduction supplementary foods after birth	≥1 Month and <6 months		39	17	56	1	
	<7 Days		40	15	55	1.16 (0.51–2.65)	0.72
	≥1 week and <1 month		22	6	28	1.59 (0.55–4.65)	0.39
	Don’t know		25	9	34		
Child diseases last two weeks	Diarrhea	Yes	24	11	35	0.55 (0.23–1.31)	0.27
		No	86	22	108		
		Unknown			30		
	Fever	Yes	44	12	56	1.15 (0.51–2.58)	0.83
		No	64	20	84		
		Unknown			33		
	Cough and respiratory issue	Yes	13	6	19	0.55 (0.19–1.6)	0.36
		No	94	24	118		
		Unknown			36		
Malaria last year	Normal		64	20	84	1	
	Malaria		48	13	61	1.15 (0.52–2.54)	0.72
	Don’t know		[14	14	28]		

*P < 0.05. HDDS: household dietary diversity score. FIES: food insecurity experience scale. MDDS-Children: minimum dietary diversity score for children.

**Table 6.** Prevalence of underweight and its odds ratio.

Independent variables			Underweight			OR (95% CI)	p-value
			Yes	No	Total (*N* = 173)		
Wealth Index	Poor		37	31	68	1	–
	Middle		20	15	35	1.117 (0.49–2.54)	0.79
	Rich		29	68	68	0.623 (0.32–1.23)	0.17
	Unknown				2		
Household assets	Radio	Yes	12	9	21	0.73 (0.29–1.84)	0.64
		No	75	77	152		
	Mobile phone	Yes	38	51	89	1.87 (1.02–3.43)	0.048*
		No	49	35	84		
	Rice mill	Yes	5	14	19	3.15 (1.08–9.17)	0.049*
		No	81	72	153		
		Unknown			1		
Non-timber forest products	<3 types		42	29	71	1	
	3–7 types		43	53	96	0.56 (0.3–1.04)	0.067
	>7 types		0	4	4	0 (0)	0.99
	Unknown				2		
	Gather insects	Yes	26	40	66	0.5 (0.27–0.94)	0.041*
		No	59	46	105		
		Unknown			2		
	Gather ant eggs	Yes	2	9	11	0.2 (0.04–0.98)	0.031*
		No	83	77	160		
		Unknown			2		
HDDS	<3 Low		11	14	25	1	
	4–6 Medium		40	39	79	1.30 (0.53–3.22)	0.56
	7–9 Good		32	28	60	1.45 (0.56–3.71)	0.43
	10–12 well		4	5	9	1.01 (0.22–4.72)	0.98
HDDS1	Cereal group	Yes	65	67	132	0.83 (0.42–1.69)	0.72
		No	22	19	41		
		Total			173		
HDDS2	White roots and tubers group	Yes	63	45	108	2.39 (1.27–4.50)	0.008*
		No	24	41	65		
		Total			173		
HDD12	Spices, condiments and pre-packed foods	Yes	66	74	140	0.51 (0.23–1.12)	0.12
		No	21	12	33		
FIES	Food security		10	7	17	1	-
	Mild		32	21	53	1.01 (0.35–3.24)	0.9
	Moderate		32	36	68	0.62 (0.21–1.82)	0.38
	Severe		13	22	35	0.41 (0.12–1.35)	0.14
MDDS-Children	<4 food groups		39	42	81		
	≥4 food groups		48	44	92	0.85 (0.47–1.54)	0.59
Breastfeeding the first one hour		Yes	82	78	160	1.68 (0.52–5.36)	0.4
		No	5	8	13		
Exclusive breastfeeding for 6 months		Yes	3	8	11	0.34 (0.09–1.34)	0.13
		No	84	77	161		
		Unknown			1		
First time introduction supplementary foods after birth	≥1 Month and <6 months		24	32	56	1	
	<7 Days		27	28	55	1.29 (0.61–2.72)	0.51
	≥1 week and <1 month		16	12	28	1.78 (0.71–4.45)	0.22
	Don’t know		20	14	34		
Child diseases last two weeks	Diarrhea	Yes	18	17	35	0.98 (0.46–2.1)	0.96
		No	56	52	108		
		Unknown			30		
	Fever	Yes	27	29	56	0.85 (0.43–1.66)	0.73
		No	44	40	84		
		Unknown			33		
	Cough and respiratory issue	Yes	6	13	19	0.39 (0.13–1.09)	0.08
		No	64	54	118		
		Unknown			36		
Malaria last year	Normal		39	45	84	1	
	Malaria		35	26	61	1.55 (0.79–3.01)	0.19
	Don’t know		13	15	28		

*P < 0.05. HDDS: household dietary diversity score. FIES: food insecurity experience scale. MDDS-Children: minimum dietary diversity score for children.

## Discussion

To plan interventions to decrease child malnutrition, it is necessary to have thorough information about the magnitude of the problem and the underlying and basic causes. Our results indicate that the prevalence of malnutrition in the remote area we studied is much more severe than national indicators would suggest. Levels of stunting were more than twice as high as the national average: it was 72.8% among the children we studied, while the LSIS II (2018) estimated 33% nationally. The rate of wasting at 10.4% was similar to the national average of 9%, but underweight which was 27% nationally and almost twice as high, 50.3%, among the children in our study. We confirmed our hypothesis that nutritional status can be more of a problem in pockets, especially chronic malnutrition. These data make an important contribution to the scarce information about these regions.

In this study, we found that many factors related to malnutrition were of interest even though only a few could be shown to be statistically significantly associated. That such a large proportion of the population scored highly on the most immediate, and underlying causes of malnutrition such as insufficient dietary intake and disease prevalence leaves no room for comparisons within the population. Also, none of the measured macro- and micronutrients were consumed sufficiently by more than half of the population. Unexpectedly, protein intake was the least insufficient, yet only 46% had consumed sufficient amounts compared to the Thai DRI. For most other nutrients, only 25% or less had sufficient intake. Closely related to dietary intake, and perhaps more concerning was that nearly all households experienced food insecurity, an underlying cause that is in many studies has been shown to be a strong predictor of (chronic) undernutrition [30]. Other indicators of also food availability scored poorly; production diversity was low and trade opportunities scarce. The distance to markets is too far to buy and sell products, given both the distance and quality of roads. For most households, Vietnamese traders travelling by motorbike are the only source of food from outside Nong district. Also, basic causes of malnutrition scored high. Nearly all households lived below the poverty line (which in Laos is 1.19 US dollar per person per day) and educational levels were very low.

The high level of chronic malnutrition was associated with a number of factors. Availability of an electric rice mill in the household was linked with a lower frequency of stunting and underweight of children, perhaps because it reduced the time women spent milling rice. From our observation in these communities, women and young girls milled rice manually in the morning and evening for about one hour, work that was completed in 5–10 minutes with an electric rice mill. Women’s autonomy in production and reduction of their workload can improve maternal and child diets and nutrition of children. Children in households having a mobile phone, as many did, were the least likely to be wasted. Having a radio appeared to increase the chances of wasting but the association was only weakly significant. Such assets are indicators of wealth as well as modes for information and communication. In rural and mountainous areas, receiving a telephone signal means closer proximity to contact with the outside world. Lao national radio frequently broadcasts policy, educational and health knowledge. A lower prevalence of wasting was found in children of families with a radio in Ghana [31].

In these communities, we found a few types of agricultural production and a dependence on NTFP to supplement food. The area has been subjected to logging and other activities that affect the environment around the villages. The food availability may become increasingly limited as a result of decreasing forest resources, non-diversified agricultural production, poor storage facilities and lack of processing [32,36]. Limited availability and accessibility of food leads to reduced dietary diversity, and we found dietary diversity to be quite low in these households. Less than half of the children reached the recommended minimum for children of four food groups. Food insecurity, especially shortage of rice, was very common in the studied area. One solution was to collect wild roots and tubers from the forest as alternative carbohydrate sources, but this practice was found in the poorest household with high food insecurity. These particular forest foods did not appear to improve the children’s nutritional status. This was also confirmed by our field observations.

However, we did not find a significant association with malnutrition, which was similar to the results reported by Ali et al. [18], based on data from three countries, Bangladesh, Ethiopia and Vietnam.

We had expected to identify more factors associated with poor nutritional status. In Vietnam, Nguyen et al. [37] identified associations between malnutrition and maternal, socio-economic and environmental factors that we did not find here. That may be related to the homogeneity of our sample. Most of the household members were poor, stunted, food insecure and produced limited foods. They were likely to have had one or more diseases regularly during the past year, considering the high numbers reporting fever and diarrhea during the past 2 weeks. Consumption patterns also led to insufficient nutrient intake, but we did not find significant associations between these inadequate intakes and malnutrition. These indicators are included in the underlying factors associated with malnutrition framework developed by UNICEF [6]. We hypothesize that even though we could not demonstrate significant associations, these factors are likely playing a very important role in the high levels of undernutrition in this setting. Access to and consumption of nutritious foods and knowledge about feeding practices are critical.

This study has several limitations. First, it was conducted in ethnic minority communities, and translation from local languages Mang-kong and Ta-oy to Lao may have led to inaccuracies. Second, anthropometric measurements may be affected by the uncertainty of the child’s date of birth; many children were born at home and lacked birth certificates. The sample size and relative homogeneity of the population made it difficult to demonstrate significant associations with the main problem of malnutrition. However, the sampling did lead to coverage of many households in this sparsely populated the study area, and the results could be considered representative for the area and possibly for other areas with similar conditions.

## Conclusion

Our results demonstrate that child malnutrition in remote highland areas of Laos is a persistent and complex problem for public health. The children’s vulnerability to malnutrition resulted from lack of adequate nutrient intake, low dietary diversity, and infectious diseases.

Nutritional interventions should include both nutrition-specific as well as nutrition-sensitive interventions. Nutrition-specific interventions should focus on micronutrient supply plans, especially for those found to be most deficient. For example, vitamin A supplementation should continue and calcium insufficiencies can be addressed by improving the supply chain to include milk and/or small fish with edible bones. In addition to nutrition-specific interventions, water, sanitation and hygiene programs and strengthening primary health care are critical to manage the frequent episodes of fever and diarrhoea, which affect nutrient uptake. Nutrition education should also be established more firmly within healthcare.

In the situation of food insecurity, alternative sources of food such as the non-timber forest products are important, although often ignored by intervention plans. Introducing natural resource management principles and preservation zones could help to maintain such food sources. Many agricultural developments focus on increasing production for sale to markets. However, in these areas with little market access, promoting nutrition-sensitive agriculture is a potential solution to increase food and nutrition security. Choosing to produce nutrient-rich crops and rearing small animals could provide macro – and micronutrients for children and family members. Nutrition education and water and sanitation interventions for mothers and children are needed to improve feeding practices and to prevent diarrheal diseases caused by unhygienic drinking water and absence of toilets. Strengthening antimalarial campaigns could also improve nutrition, as many children in these areas have had malaria. Women and mothers play important roles in gathering water, firewood, foods, and child care, so reducing women’s workloads could also improve not only their quality of life but also their children’s health. The selected interventions should be acceptable and sustainable at the village level and linked to what is possible in local agriculture. Solutions should involve collaboration among health and agriculture sectors at least and other sectors that can contribute.

## Supplementary Material

Supplemental Material

## Paper context

Based on field reports and the scarce scientific evidence, in areas with mostly ethnic minority subsistence farming communities in Lao PDR malnutrition is much higher than the averages provided by national statistics. These communities seldom benefit from national policies targeting nutrition, and have been left behind in socio-economic development. Therefore, this study provides insights into the prevalence and the factors associated with malnutrition among upland ethnic minorities. This evidence should inform and contextualize policies and interventions to reduce malnutrition not only in Laos but in other similar communities in other countries.
