# Supplementary material for: Factors associated with child malnutrition in mountainous ethnic minority communities in Lao PDR
Source: Glob Health Action. 2020 Aug 3;13(Suppl 2):1785736. doi: 10.1080/16549716.2020.1785736 (PMC7480462; doi:10.1080/16549716.2020.1785736)
Supplement: Supplemental Material [file ZGHA_A_1785736_SM1915.docx]

**Supplementary materials**

Additional file 1. Tool 2 Household questionnaire

Additional file 2. Tool 3 Woman questionnaire

Additional file 3. Tool 5. Children under five questionnaire

Additional file 9. Tool 9. 24-hour recall questionnaire
